# Genome-wide association studies have problems due to confounding: Are family-based designs the answer?

**DOI:** 10.1371/journal.pbio.3002568

**Published:** 2024-04-12

**Authors:** Alexander Strudwick Young

**Affiliations:** 1 UCLA Anderson School of Management, Los Angeles, California, United States of America; 2 Human Genetics Department, UCLA David Geffen School of Medicine, Los Angeles, California, United States of America

## Abstract

Genome-wide association studies (GWASs) can be affected by confounding, but family-based GWASs use random, within-family genetic variation to avoid this. This Primer explores a study in PLOS Biology which asks how different sources of confounding affect GWASs and whether family-based designs offer a solution.

## Teaser

Genome-wide association studies (GWASs) can be affected by confounding. Family-based GWAS uses random, within-family genetic variation to avoid this. A study in *PLOS Biology* details how different sources of confounding affect GWAS and whether family-based designs offer a solution.

Since the advent of large-scale, genome-wide genotype data, one study design has dominated human genetics: the genome-wide association study (GWAS). A GWAS scans across the genome for loci that are associated with a phenotype. However, GWASs are susceptible to confounding due to gene–environment correlation (environmental confounding) and correlations with other genetic variants across the genome (genetic confounding). Although GWASs use techniques including principal component analysis (PCA) to adjust for population structure, this often fails to eliminate all confounding [1–3]. The confounding present in GWAS has caused issues in downstream applications, including: estimation of heritability and genetic correlation [4,5], estimation of disease causes using mendelian randomization [6], and inferences of natural selection [2]. Family-based GWAS (FGWAS) has been proposed as a solution to confounding because genotypes of offspring are randomly assigned given the genotypes of the parents, generating a natural experiment. Recent FGWASs have demonstrated confounding in GWASs of several phenotypes [7,8], including educational attainment, cognitive ability, height, smoking, and age when first giving birth. In this issue of *PLOS Biology*, Veller and Coop [9] perform a comprehensive evaluation of how different phenomena can lead to bias in GWAS and FGWAS, finding that FGWAS is free from all environmental confounding and almost all genetic confounding.

Typically, GWASs analyze one focal variant at a time, so GWAS associations include the direct genetic effects (DGEs, causal effects of alleles in an individual on that individual) of the focal variant and variants that are correlated with the focal variant (i.e., in linkage disequilibrium or LD). Variants that are physically close (on the same chromosome) to the focal variant tend to be in strong LD as they tend to be inherited from the same parental haplotype without recombination. This makes it hard to pinpoint the causal variant, leading to the problem of fine-mapping. Since this occurs even under random-mating, it’s not typically thought of as genetic confounding even though it leads to genotype–phenotype association for non-causal variants. Furthermore, many methods are designed to work with this type of local LD that always affects GWAS.

**Fig 1:**
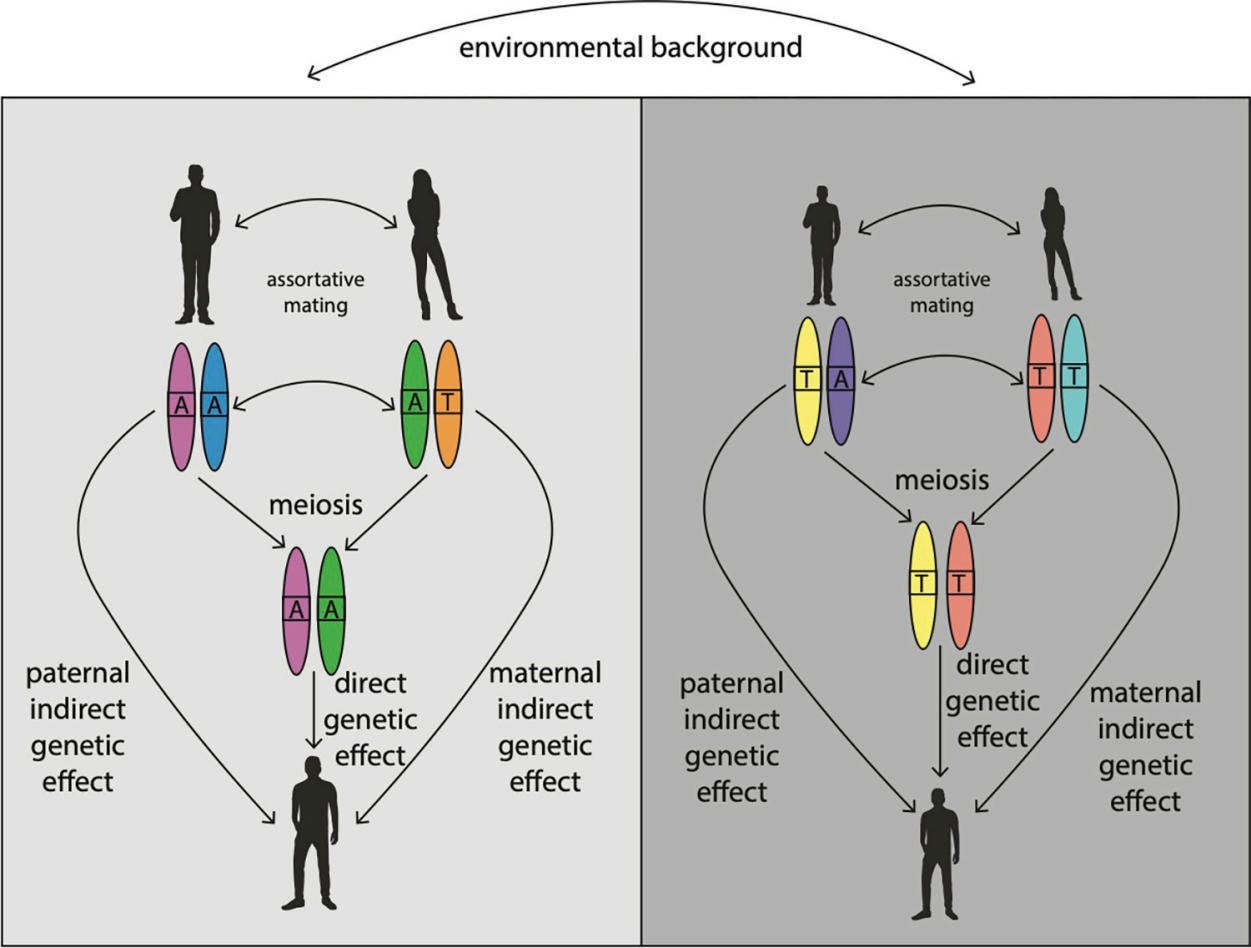
Sources of genotype–phenotype association. The family on the left has a higher frequency of the A allele and is taller than the family on the right. This could lead to a GWAS finding that the A allele is associated with increased height. However, several distinct phenomena could contribute to the GWAS association. If the A allele exerts a different effect on the bodies of those carrying it compared to the T allele, leading to increased height, this would contribute to genotype–phenotype association and be an example of a DGE. However, if the A allele was inherited from a parent, the A allele could have affected the offspring’s height through another pathway: by affecting the parent’s phenotype (e.g., nurturing behavior) and thereby affecting the offspring’s height through the environment, an example of an IGE. IGEs can be considered as confounding factors when the goal is estimation of DGEs—implicitly the goal of most GWASs. However, genotype–phenotype associations can occur at loci without any causal influence on the phenotype due to population stratification, which occurs when there is a correlation between allele frequencies and environments across genetically distinct subpopulations. For example, the family on the left could be from a subpopulation with abundant calories and a high frequency of the A allele, whereas the family on the right could be from a subpopulation with insufficient calories and a low frequency of the A allele; this could generate a spurious association between the A allele and increased height. Another source of confounding is assortative mating, which results in correlation between mates’ phenotypes, and therefore between the causal alleles in the mother and the father, irrespective of their physical positions, inducing genetic confounding between the focal A allele and all other causal alleles in the genome. Family-based GWAS (FGWAS) exploits the randomization of genetic material during meiosis to remove confounding from estimates of DGEs.

However, phenomena other than DGEs of the focal and nearby variants can contribute to the genotype–phenotype associations picked up by GWAS [3] (Fig 1): indirect genetic effects (IGEs, effects of alleles in an individual on another individual mediated through the environment) from relatives (e.g., parents or siblings) contribute to genotype–phenotype associations and cannot—in general—be removed without data on first-degree relatives of the GWAS sample. Although IGEs are a form of gene–environment correlation, they do not require the population to be structured. When there is population structure, this can lead to correlations between alleles and other environment factors (called population stratification, an example of environmental confounding). For example, two reproductively isolated populations could have different rates of skin cancer due to living at different latitudes: this could lead to a spurious associations (in the overall population) between alleles that are at different frequencies in these two populations and skin cancer risk. While some forms of population structure can be corrected for using PCA and other methods, subtle forms of stratification are difficult to detect and remove from GWAS [1].

Nonlocal genetic confounding—including across chromosomes—can result from nonrandom mating, including population structure, natural selection, and assortative mating. Assortative mating leads to correlations between mates’ phenotypes and therefore between the trait increasing alleles in the mother and the father, irrespective of their physical positions [10]. Over multiple generations of assortative mating, alleles that have the same direction of effect become positively correlated both within haplotypes from the same parent and across haplotypes from different parents. GWAS will therefore overestimate the DGE of a causal variant for a trait affected by (positive) assortative mating because it picks up part of the effect of all other causal variants, including rare variants and variants on other chromosomes [10]. The picture is further complicated by the fact that humans do not assort on a single phenotype, but on multiple dimensions involving multiple phenotypes simultaneously, inducing correlations across genetic variants affecting different traits [5].

A potential solution to the confounding that can affect GWAS is to instead perform FGWAS, which uses within-family genotype variation to estimate DGEs. Within-family variation is generated by random segregations of chromosomes during meiosis, which are independent of environment; thus, FGWAS eliminates confounding due to gene–environment correlation (including IGEs from parents and population stratification).

However, exactly how genetic confounding affects FGWAS had not been thoroughly investigated until the work of Veller and Coop [9]. Because chromosomes segregate independently during meiosis, genetic confounding from variants on different chromosomes does not affect FGWAS. Within-chromosome, FGWAS still has to contend with the fine-mapping problem due to a lack of recombination between nearby variants. What was not clear until Veller and Coop’s study was how FGWAS is affected by genetic confounding due to variants on the same chromosome but not in the same local region (LD block) as the focal variant. They examine theoretically and in simulations how both GWAS and FGWAS are affected by assortative mating, population structure (including due to phenotype-based migration), admixture, and natural selection (in the form of stabilizing selection). They find that all the sources of genetic confounding can lead to substantial bias in GWAS, but that the confounding in FGWAS is generally minimal. This is because the human genome is split over 23 chromosomes, implying that most pairs of loci are on different chromosomes. If traits are affected by many causal variants spread across the genome, then there is much less potential for genetic confounding due to correlations between variants on the same chromosome than due to all genome-wide variants. Since FGWAS eliminates the influence of cross-chromosome correlations, the vast majority of genetic confounding is eliminated. However, Veller and Coop show that a small amount of (nonlocal, but within chromosome) genetic confounding can remain under certain scenarios.

Veller and Coop also give warnings about interpreting coefficients on parental genotypes as estimates of IGEs—as they are affected by both genetic and environmental confounding—and on interpreting the results of within-family polygenic prediction analyses. Polygenic predictors (called polygenic scores or PGSs) are weighted sums of genotypes, with weights typically derived from GWAS. Within-family association between a PGS and phenotype can only be due to DGEs (and IGEs between siblings if using a sibling design [7]) but can be misinterpreted when there is assortative mating on multiple traits. This could lead to a PGS for one trait predicting another trait within-family, despite there being no shared causal variants. This can occur because the PGS for one trait could give nonzero weight to variants that are causal for the other trait due to correlations between variants for both traits induced by assortative mating. This concern would be practically eliminated for within-family associations with PGSs derived from FGWAS, but these are currently far less powerful than PGS derived from GWAS [8].

GWAS has successfully discovered thousands of trait–variant associations, given biological insights, guided drug target discovery, and enabled creation of powerful polygenic predictors. However, GWAS is susceptible to confounding that can lead to biases and erroneous conclusions in downstream analyses. FGWAS presents a principled solution by using the natural experiment of mendelian segregation during meiosis to remove confounding. Veller and Coop examine FGWAS and find that it removes all environmental confounding and almost all genetic confounding. Future studies should examine the consequences of the phenomena investigated by Veller and Coop on downstream applications of both GWAS and FGWAS (e.g., estimating genetic correlations and natural selection). While FGWAS has favorable properties compared to GWAS, it requires samples with genotyped first-degree relatives, which limits the sample size compared to GWAS: existing FGWASs have effective sample sizes in the tens of thousands [7,8], compared to millions for many GWASs. Even with comparable sample sizes, FGWAS is less powerful than GWAS because it only uses within-family genetic variation. Furthermore, issues not examined by Veller and Coop may lead to bias in FGWAS, such as nonrandom sampling of offspring with respect to heritable phenotypes. We should therefore consider building family-based sampling (ideally representative of the population) into the design of future biobanks to enable powerful and robust FGWAS and other family-based methodologies.
